# Mediterranean Diet and Quality of Life in Adults: A Systematic Review

**DOI:** 10.3390/nu17030577

**Published:** 2025-02-05

**Authors:** Justyna Godos, Monica Guglielmetti, Cinzia Ferraris, Evelyn Frias-Toral, Irma Domínguez Azpíroz, Vivian Lipari, Andrea Di Mauro, Fabrizio Furnari, Sabrina Castellano, Fabio Galvano, Licia Iacoviello, Marialaura Bonaccio, Giuseppe Grosso

**Affiliations:** 1 Department of Biomedical and Biotechnological Sciences, University of Catania, 95123 Catania, Italy; 2 Center for Human Nutrition and Mediterranean Foods (NUTREA), University of Catania, 95123 Catania, Italy; 3 Human Nutrition and Eating Disorder Research Center, Department of Public Health, Experimental and Forensic Medicine, University of Pavia, 27100 Pavia, Italy; 4 Laboratory of Food Education and Sport Nutrition, Department of Public Health, Experimental and Forensic Medicine, University of Pavia, 27100 Pavia, Italy; 5 School of Medicine, Universidad Espíritu Santo, Samborondón 0901952, Ecuador; 6 Research Group on Food, Nutritional Biochemistry and Health, Universidad Europea del Atlántico, Isabel Torres 21, 39011 Santander, Spain; 7 Universidade Internacional do Cuanza, Cuito EN250, Angola; 8 Universidad de La Romana, La Romana 22000, Dominican Republic; 9 Universidad Internacional Iberoamericana, Campeche 24560, Mexico; 10 Department of Educational Sciences, University of Catania, 95124 Catania, Italy; 11 Research Unit of Epidemiology and Prevention, IRCCS Istituto Neurologico Mediterraneo NEUROMED, 86077 Pozzilli, Italy; 12 Department of Medicine and Surgery, LUM University, 70010 Casamassima, Italy

**Keywords:** Mediterranean diet, quality of life, aging, frailty, physical resilience, systematic review

## Abstract

Background/Objectives: With the increasing life expectancy and, as a result, the aging of the global population, there has been a rise in the prevalence of chronic conditions, which can significantly impact individuals’ health-related quality of life, a multidimensional concept that comprises an individual’s physical, mental, and social wellbeing. While a balanced, nutrient-dense diet, such as Mediterranean diet, is widely recognized for its role in chronic disease prevention, particularly in reducing the risk of cardiovascular diseases and certain cancers, its potential benefits extend beyond these well-known effects, showing promise in improving physical and mental wellbeing, and promoting health-related quality of life. Methods: A systematic search of the scientific literature in electronic databases (Pubmed/Medline) was performed to identify potentially eligible studies reporting on the relation between adherence to the Mediterranean diet and health-related quality of life, published up to December 2024. Results: A total of 28 studies were included in this systematic review, comprising 13 studies conducted among the general population and 15 studies involving various types of patients. Overall, most studies showed a significant association between adherence to the Mediterranean diet and HRQoL, with the most significant results retrieved for physical domains of quality of life, suggesting that diet seems to play a relevant role in both the general population and people affected by chronic conditions with an inflammatory basis. Conclusions: Adherence to the Mediterranean diet provides significant benefits in preventing and managing various chronic diseases commonly associated with aging populations. Furthermore, it enhances the overall health and quality of life of aging individuals, ultimately supporting more effective and less invasive treatment approaches for chronic diseases.

## 1. Introduction

Health-related quality of life (HRQoL) is a multidimensional concept that encompasses an individual’s physical, mental, and social wellbeing, as influenced by their health status and healthcare experiences [1]. Over the past few decades, HRQoL has gained increasing prominence as an essential measure in public health, clinical research, and healthcare policy [2]. It offers a comprehensive understanding of how health conditions and interventions impact daily living and overall life satisfaction, particularly for adults and older individuals [3]. Given the aging global population and the rising prevalence of chronic conditions, HRQoL in these age groups has become a critical area of research, providing insights that inform healthcare strategies and improve patient-centered care [4]. In adults and older individuals, the determinants of HRQoL are numerous and complex, ranging from physiological and psychological factors to environmental and social influences [5]. Chronic diseases, such as cardiovascular disorders, diabetes, arthritis, and respiratory conditions are common in the growing older populations and can severely impact their ability to perform everyday activities, manage symptoms, and maintain a high quality of life [6,7,8,9]. Moreover, the age-related decline in physical function, cognitive impairment, and the onset of multimorbidity further exacerbate HRQoL concerns [10]. These challenges, coupled with social isolation, financial constraints, and limited access to healthcare, underscore the need for a holistic understanding of how health, wellbeing, and life satisfaction interact and how HRQoL would affect lifespan and mortality [11]. Research into HRQoL has expanded beyond traditional medical paradigms to include psychological and social perspectives, emphasizing the importance of mental health and social connectivity in shaping overall wellbeing [12,13]. For older adults, maintaining cognitive function, emotional stability, and social participation are crucial factors that influence HRQoL [14,15]. Studies have shown that interventions aimed at improving mental health, encouraging physical activity, and fostering social support networks can lead to significant improvements in HRQoL among older adults [16,17].

The Mediterranean diet has been widely investigated as a healthy dietary model characterized by the high consumption of fruits, vegetables, whole grains, legumes, nuts, olive oil and alternate sources of protein, such as poultry, fish, eggs, and dairy, with moderation concerning meat and red wine (during meals), alongside a variety of peculiar features, such as the use of spices, food variety and moderation, preference for locally produced foods, the promotion of conviviality and specific culinary approaches [18]. All these features ensure a balanced diet that provides essential nutrients, such as healthy fats, adequate amounts of protein, and complex carbohydrates, vitamins and minerals, as well as a variety of bioactive compounds, including (poly)phenols [19]. A balanced, nutrient-dense diet can significantly promote physical and mental wellbeing by modulating the gut microbiota and the immune system [20]. Diets rich in fruits, vegetables, and whole grains can have immune-regulatory properties that help to counteract oxidative stress, a key driver of aging and chronic disease [21]. These benefits may, in part, result from bioactive molecules, such as (poly)phenols [22]. Inflammatory processes, which are often exacerbated in older adults due to both aging and the presence of comorbid conditions, can also be mitigated by anti-inflammatory foods, including those high in omega-3 polyunsaturated fatty acids (PUFA), such as fish, nuts, and seeds [23]. These foods help regulate the body’s immune response and may reduce the risk of frailty, muscle loss, and cognitive decline. Moreover, a healthy diet can help regulate blood sugar and improve insulin sensitivity, which is particularly important for individuals with or at risk of developing type-2 diabetes mellitus (T2DM) or metabolic syndrome [24].

Although reported to be slowly losing popularity in Mediterranean regions [25], the Mediterranean diet is widely recognized for its role in disease prevention, particularly in reducing the risk of cardiovascular diseases and certain cancers [26,27]. Moreover, adherence to the Mediterranean diet has demonstrated beneficial effects in the secondary prevention of conditions related to metabolic and cardiovascular complications [28]. Also, the occurrence of certain cancers (i.e., colorectal and breast cancer) have been inversely associated with adherence to the Mediterranean diet [29,30]. Moreover, the adoption of such a dietary pattern may also be associated with better health status in secondary preventions [31]. Hence, the potential benefits of the Mediterranean diet extend beyond these well-known effects, showing promise in improving the health and quality of life in individuals affected by chronic diseases other than cardiovascular diseases and cancer, particularly in aging populations. In fact, a growing body of evidence suggests that the Mediterranean diet may exert therapeutic effects on a variety of health conditions commonly seen in the elderly, such as neurodegenerative conditions, affective disorders, and musculoskeletal diseases [32,33,34]. The diet’s anti-inflammatory, antioxidative, and metabolic-regulating properties are proposed as the mechanisms underlying these benefits, which could be critical in enhancing health outcomes and slowing the progression of these diseases [19].

In light of these considerations, understanding whether the scientific literature supports the evidence that higher adherence to the Mediterranean diet would be associated with higher HRQoL could be interesting in exploring the possibility of implementing diet into strategies to maintain or restore health status in the aging populations. A previous overview of the literature included the Mediterranean diet among various dietary patterns associated with better HRQoL, but results were limited to few reports and not specifically focused on the main features associated with this dietary pattern [35]. The aim of this article is to review the current literature on the relation between adherence to the Mediterranean diet and HRQoL in the adult population. By highlighting the complex interactions between health, aging, and life satisfaction, this paper aims to provide a comprehensive overview of HRQoL and its implications for healthcare practice and policy.

## 2. Materials and Methods

The design and reporting of this study followed the Meta-analyses of Observational Studies in Epidemiology (MOOSE) guidelines (Table S1) [36]. The systematic review protocol was registered in the PROSPERO International Prospective Register of Systematic Reviews database (ID: CRD42024627566, at https://www.crd.york.ac.uk/prospero/ accessed on 30 January 2025).

### 2.1. Search Strategy and Study Selection

A systematic search of the scientific literature in electronic databases (Pubmed/Medline) was performed to identify potentially eligible studies published up to January 2024. The search strategy was based on the combination of the relevant keywords imputed as text words, including “Mediterranean diet” and “quality of life” (Table S2). The eligibility of the studies for the systematic review was assessed using the PICOS approach (Table S3) according to the following criteria, focusing on studies that: (i) were conducted on adults (i.e., mean age > 18 years old); (ii) had an observational design (cohort studies, cross-sectional studies, case–control studies); (iii) assessed food intake and the level of adherence to the Mediterranean diet through either 24 h recalls, food frequency questionnaires (FFQ), or dietary diaries and validated instruments, respectively; (iv) assessed quality of life through validated instruments. Only English language studies were considered for full text evaluation. The reference lists of all eligible studies were also examined for any additional studies not previously identified. The systematic literature search and study selection were performed by two independent authors (J.G. and G.G.), and any incongruity was resolved through a discussion and reaching consensus.

### 2.2. Data Extraction and Quality Assessment

Data from all eligible studies were extracted using a standardized electronic form. The following information was collected: first author name, publication year, study design and location, population age and sex, sample size, health status, details on the assessment method of dietary habits, adherence to the Mediterranean diet and quality of life, and the main findings of the study. The quality of each eligible study was evaluated using the Newcastle–Ottawa Quality Assessment Scale [37], a scoring system based on 3 main domains of quality (selection, comparability, and outcome) assessing specific study characteristics depending on the type of study design with a scoring over 5 and 7 points for cross-sectional and prospective studies, respectively, identified as being of good/high quality. Two investigators extracted the data (A.D.M. and F.F.) and assessed the methodological quality independently, and any incongruity was reviewed by a third author (G.G.) and resolved through a discussion by reaching consensus.

## 3. Results

### 3.1. Overview of the Study Selection

**Figure 1:**
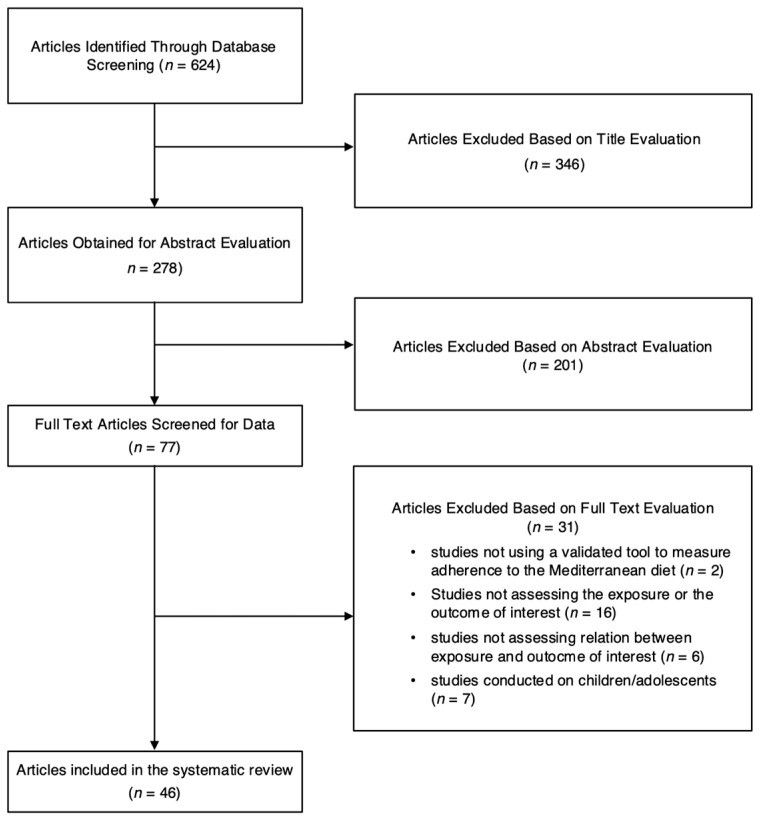
Flowchart of study selection process.

A summary of the study selection is shown in Figure 1. A total of 624 studies were considered of potential interest for this systematic search. Following title and abstract evaluation, 547 studies were excluded and 77 studies were selected for full-text evaluation. After the exclusion of 31 studies, a total of 46 studies were included in this systematic review [38,39,40,41,42,43,44,45,46,47,48,49,50,51,52,53,54,55,56,57,58,59,60,61,62,63,64,65,66,67,68,69,70,71,72,73,74,75,76,77,78,79,80,81,82,83].

### 3.2. Characteristics of the Studies Included

A total of 13 studies were conducted in the general population, while 15 studies involved various types of patients. Out of the total studies reviewed, the majority had a cross-sectional/case-control design and eight were prospective. The number of participants varied between a few dozens, especially in more clinical studies, to up to nearly 17,000 in more epidemiological ones. Most studies used food frequency questionnaires (FFQs) to detect dietary habits, although some studies have resorted also to the use of food diaries and 24 h recalls. There were a variety of tools used to assess the level of adherence to the Mediterranean diet, including the Mediterranean diet score (MDS), the Mediterranean Diet Adherence Screener (MEDAS) used in the PREDIMED trial, the relative Mediterranean Diet Score (rMED), the alternate Mediterranean Diet score (aMED), and the literature-based Medi-Lite score. Similarly, a large variety of validated instruments have been employed to measure the quality of life in both the general population and patients, including the SF-36 and SF12 as the most common, and some specific tools used for individual diseases (i.e., the Audit of Diabetes-Dependent Quality of life (ADDQoL-19), the Dermatology Life Quality Index (DLQI), the Asthma Quality of Life Questionnaire (AQLQ), the Kidney Disease Quality of Life Short Form (KDQOL-SF), the Inflammatory Bowel Disease Questionnaire (IBDQ), the Crohn’s and Ulcerative Colitis Questionnaire (CUCQ-8), and the Multiple Sclerosis Quality of Life-54 (MSQOL-54)). In general, almost all studies reported a significant association between adherence to the Mediterranean diet and better HRQoL, although no specific variables were identified as barriers or facilitators to such relation. When considering study quality, the majority of cross-sectional studies were scored as good or high quality, case–control studies were scored as good quality, while all prospective studies were scored as high quality (Tables S4–S6).

### 3.3. Details of the Main Results of the Studies Included: General Population

The main characteristics of the studies included in the systematic review reporting on the association between adherence to the Mediterranean diet and quality of life among the general population are reported in Table 1. Among studies conducted on the general population, the earliest studies were conducted in Spain. The first one, including two population-based cross-sectional surveys conducted in Gerona (Spain) involving 3054 randomly selected free-living men and women (25–74 y) and 6322 men and women (35–88 y), using the SF-12 to assess HRQoL and the MDS to assess the adherence to the Mediterranean diet, showed a significant association of the latter with both the physical and mental components of the tool in both men (β = 0.160, 95% CI: 0.076–0.246 and β = 0.148, 95% CI: 0.035–0.261, respectively), and only the physical component in women (β = 0.230, 95% CI: 0.105–0.356) [38]. The second was a prospective analysis on the SUN (Seguimiento Universidad de Navarra) cohort involving 11,015 participants with 4 years of follow-up investigating the relation between the adoption of the Mediterranean diet (retrieved from a 136-item FFQ) and HRQoL assessed through the SF-36 Health Survey, reporting a significant association between higher adherence to this dietary pattern and most mental health domains (vitality, social functioning and role emotional), with vitality (β = 0.50, 95% CI: 0.32, 0.68) and general health (β = 0.45, 95% CI: 0.26, 0.62) showing the highest coefficients. Besides this, physical functioning, with physical bodily pain, general health and vitality domains were significantly better with increasing adherence to the Mediterranean diet [39]. Following this study, a number of cross-sectional investigations conducted in the Mediterranean region contributed to corroborating the retrieved associations between adherence to the Mediterranean diet and HRQoL.

Among studies conducted in Spain involving a relatively small sample, a cross-sectional study on 351 older adults (>60 y) aimed to evaluate the relationship between adherence to the Mediterranean diet and HRQoL and degree of life satisfaction among older adults. An 11-item MEDIS-FFQ was used in order to ascertain the dietary habits, meanwhile a 9-item MDS was calculated to estimate the adherence to the Mediterranean diet. Furthermore, we used the SF-12 questionnaire to assess the HRQoL and the Satisfaction with Life Scale (SWLS) to calculate life satisfaction. Participants with higher adherence to the Mediterranean diet engaged in more physical activity (*p* = 0.01), reported better HRQoL (*p* < 0.05), and consumed fewer alcoholic beverages (*p* = 0.04). In models adjusted for age, significant associations were observed between Mediterranean diet adherence and mental function in both sexes, while physical function showed a significant link only in men. In fully adjusted models, a positive relationship was identified between Mediterranean diet adherence and life satisfaction among women (*p* > 0.05), but no such association was found for men (*p* = 0.31). Adherence to the Mediterranean diet is positively linked to self-reported physical and mental function in both male and female and to life satisfaction among women [40]. Also, another cross-sectional study conducted in Spain including 127 university professors investigated the relationship between adherence to the Mediterranean diet, HRQoL and anthropometric measurements. To estimate adherence to the Mediterranean diet, we used the 14-item MEDAS questionnaire, while HRQoL was measured using the SF-36. The mean PREDIMED score was 8.29 ± 1.77, with 48.8% of participants classified as having high adherence (≥9 points). Men showed higher mean weight, BMI and waist/hip ratio (WHR), whereas women exhibited a higher fat percentage (30.9% vs. 27.5%; *p* = 0.02). A significant negative correlation was found between MD adherence and BMI (r = −0.179; *p* < 0.05) and fat percentage (r = −0.228; *p* < 0.05). Mediterranean diet adherence showed a significant positive relationship with vitality (r = 0.233; *p* = 0.009), social functioning (r = 0.229; *p* = 0.008) and the mental component summary (r = 0.205; *p* = 0.021). The regression analysis identified the mental component summary (β = 0.239, *p* = 0.041), diastolic pressure (β = −0.473, *p* < 0.001), and fat percentage (β = −0.241, *p* = 0.004) as significant predictors of Mediterranean diet adherence [41]. From the same group of researchers, another study involving both professors (n = 127) and students (n = 272) confirmed only the relation between adherence to the Mediterranean diet and the physical component of HRQoL (β = 0.03, *p* = 0.03) [42]. The inclusion of students (i.e., younger individuals) in the sample led to less significant results, suggesting that the retrieved association between higher adherence to the Mediterranean diet and better HRQoL may be more relevant among older individuals. A cross-sectional study, conducted in Spain and aimed at examining how adherence to the Mediterranean diet influences HRQoL in 520 participants aged 41–80 years old, showed that in older individuals (71–80 age group), higher Mediterranean diet adherence was significantly correlated with a better physical component of the SF-36 questionnaire (r = 0.367, *p* = 0.014). Significant differences between age groups were observed only in fruit (*p* < 0.001) and fish (*p* = 0.002) consumption, with the highest consumption percentages recorded in the 71–80 age group (56.82% for fruit and 52.27% for fish) [43]. Lately, a large observational cross-sectional study that included 17,333 adults (with various cardiometabolic health statuses) from the NutrIMDEA Study, an online Spanish survey, was performed to investigate the relationships of sociodemographic variables, anthropometric data, dietary habits and lifestyle factors with HRQoL evaluated using the SF-12 survey. A higher score for Mediterranean diet adherence (assessed using the 14-item PREDIMED questionnaire) and better PCS12 score were observed in the participants with healthy cardiometabolic status, unlike in the diseased ones (7.6 ± 2.1 vs. 7.4 ± 2.2, *p* < 0.001 and 54.5 ± 6.2 vs. 51.0 ± 8.0, *p* < 0.001). Additionally, greater Mediterranean diet adherence was observed in participants with very good health compared to those with poor/fair HRQoL (8.0 ± 2.1 vs. 6.7 ± 2.1, *p* < 0.001). European/Caucasian subjects showed a greater Mediterranean diet adherence score and better PCS12 score (7.7 ± 2.1 vs. 7.2 ± 2.6, *p* < 0.001; 53.7 ± 6.8 vs. 53.0 ± 7.1, *p* < 0.001) than participants with other ethnicities [44]. Among studies conducted in Spain, one study [45] provided longitudinal analyses concerning HRQoL in relation to adherence to the Mediterranean diet. The study included 15,674 participants of the SUN cohort followed-up for about 4 years. The authors showed that Mediterranean diet was associated with all components of the SF-36 HRQoL questionnaire (global, β = 0.32, 95% CI—0.22–0.42; physical, β = 0.37, 95% CI—0.25–0.50; mental, β = 0.27, 95% CI—0.16–0.37; β = 0.39, 95% CI—0.25–0.54) [45].

Another group of studies have been conducted in Italy. A cross-sectional study involving a population of 16,937 participants (>35 y) from the general population participating in the Moli-sani study aimed to highlight if there was an association between the Mediterranean diet and HRQoL, and to assess the roles of dietary antioxidants, fiber content and fatty acid components. Adherence to the Mediterranean diet was tested through a 188-item FFQ, an Italian Mediterranean Index (IMI), the MDS, and by principal component analysis (PCA), while HRQoL was assessed by the SF-36 Health Survey. A significant association was revealed for MDS, IMI and an “olive oil and vegetable” pattern (PCA1), specifically, with mental health; a β = 0.36 (95% CI: 0.21–0.51) was derived for IMI and β = 0.33 (95% CI: 0.18–0.48) for MDS, while the “eggs and sweets” pattern (PCA3) showed no significant association with mental health. There was a positive association between physical health and MDS (β = 0.15, 95% CI: 0.06–0.24) and PCA1 (β = 0.15; 95% CI: 0.06–0.24), while a negative association was observed with the “meat and pasta” pattern (β = −0.11, *p* < 0.05). Participants with the highest Mediterranean diet adherence had 42% (MDS), 34% (IMI) or 59% (PCA1) statistically significant multivariable odds of being in the uppermost level of mental health, as compared with subjects in the lowest category. However, these associations disappeared after adjusting for total food antioxidant content or dietary fiber, while they remained not influenced by the inclusion of either monounsaturated or polyunsaturated fatty acids. Significantly higher odds of being in the top level of physical health were seen in subjects in the highest PCA1 or PCA3 [46]. Another study conducted in the Italian general population involved 2044 adults from southern Italy. Dietary habits and adherence to the Mediterranean diet were assessed through the administration of a 110-item FFQ and the application of the Medi-Lite score. The MANSA was used to assess self-rated HRQoL. Total and individual component scores of the HRQoL tool (with the exception of mental health) were individually linearly associated with the Mediterranean diet adherence score [47]. From the same cohort, a subsample of 883 older participants (>50 y) was investigated for HRQoL in the context of successful aging. The multivariate analysis showed that higher adherence to the Mediterranean diet was significantly associated with HRQoL (OR = 14.04, 95% CI: 6.81–28.93) as well as cognitive impairment, depressive symptoms, sleep quality, and overall successful aging [48].

Among the studies conducted in the Mediterranean countries, the Mediterranean Diet in Older Adults (MINOA) study analyzed data collected from 436 adults (>65 y), both male and female, living in Greece. The study aimed to explore the relationships between Mediterranean diet adherence, social capital, and HRQoL. Dietary habits were assessed using an 11-item FFQ, while adherence to the Mediterranean diet was evaluated with the MDS. HRQoL was measured via the SF-36 survey, providing scores for physical and mental health domains. Multivariate linear regression analyses demonstrated that both social capital and HRQoL positively influenced Mediterranean diet adherence, with these effects remaining significant after adjusting for confounders and acting independently of each other. Specifically, the total social capital and its value of life/social agency component showed positive associations with Mediterranean diet adherence (respectively, β = 0.04 and β = 0.1, *p* < 0.05). Regarding HRQoL, only the physical health components exhibited a significant positive relationship with the Mediterranean diet adherence score (β = 0.09, *p* < 0.001). Furthermore, total social capital was strongly associated with perceived physical and mental health (β = 0.21 and β = 0.28, *p* < 0.001, respectively). In conclusion, these findings suggest that in older adults, social capital, HRQoL and Mediterranean diet adherence are interconnected, influencing both dietary quality and overall quality of life [49]. Another study conducted in Greece involved 3254 community-dwelling Greek older people (aged >65 y) tested for adherence to the Mediterranean diet through the 11-item MDS and HRQoL with the SF-36 tool. The study showed that high adherence to the Mediterranean diet was independently associated with better HRQoL (OR: 2.31, 95% CI: 2.06–2.68) alongside higher physical activity levels and better sleep quality [50]. Finally, a cross-sectional study conducted in Greece during the COVID-19 pandemic evaluated the relationship between Mediterranean diet adherence and multiple sociodemographic, anthropometric and lifestyle factors among 3721 adults aged 18–65 years (mean age 37.6 ± 5.8). Participants completed validated questionnaires such as World Health Organization Quality of life Brief version (WHOQOL-BREF) for assessing the quality of life of participants, among others, and the 11-item Mediterranean Diet Score in order to assess the adherence to the Mediterranean diet. Participants with high Mediterranean diet adherence exhibited better quality of life (65.1%, *p* = 0.001); in the multivariate analysis, Mediterranean diet adherence was associated with better quality of life, irrespective of other factors (OR = 2.04, 95% CI—1.83–2.27, *p* = 0.0098), and lower odds of anxiety (OR = 2.18, *p* = 0.0107) and depression (OR = 2.43, *p* = 0.0045) [51].

Some studies also investigated the potential association between higher adherence to the Mediterranean diet and HRQoL outside the Mediterranean basin. A prospective study intended to investigate the relationship between Mediterranean diet adherence and its specific dietary components with the HRQoL in pregnant women from Spain and Sweden. The Spanish cohort, which was part of a project called GESTAFIT, included 138 females, while the Swedish cohort, deriving from the HealthyMoms trial, included 302 females. To evaluate dietary habits, the 78-item FFQ for the Spanish participants and the 3-day 24 h recalls for the Swedish participants were used, while Mediterranean diet adherence was assessed using an 8-item MDS at the 14–16th gestational weeks. HRQoL was calculated with the Spanish and Swedish versions of the SF-36 or RAND-36 at the 14–16th and 34–37th gestational weeks. In the Spanish sample, higher Mediterranean diet adherence correlated significantly with better physical functioning (β = 0.183, *p* = 0.038), vitality (β = 0.190, *p* = 0.031), emotional role (β = 0.191, *p* = 0.032), and mental health (β = 0.176, *p* = 0.049) during the second trimester. Longitudinal analyses showed that higher Mediterranean diet adherence was consistently associated with lower bodily pain in both cohorts during the third trimester (Spanish cohort β = 0.215, *p* = 0.016; Swedish cohort β = 0.186, *p* = 0.005). This association seems to be explained by the greater consumption of fiber (β = 0.172–0.173, *p* < 0.05), fruits (β = 0.202–0.252, *p* < 0.01), fish (Spanish cohort β = 0.228, *p* = 0.020), nuts (Swedish cohort β = 0.165, *p* = 0.011) and legumes (Swedish cohort β = 0.155, *p* = 0.049) [52]. Another prospective study conducted in Australia in the context of the Wellbeing, Eating and Exercise for a Long Life (WELL) study involved 2457 adults (55–65 y) evaluating dietary habits through a 111-item FFQ and the application of an 8-item MDS (with the exclusion of olive oil due to missing data from the FFQ) to assess the adherence to the Mediterranean diet (alongside other diet quality scores). HRQoL was assessed through the RAND 36. In general, higher adherence to the Mediterranean diet was associated with better physical function (highest quartile, OR = 1.26, 95% CI—1.00–1.60), general health (highest quartile, OR = 1.31, 95% CI—1.04–1.65), and energy (highest quartile, OR = 1.39, 95% CI—1.10–1.75); compared to the lowest quartile, individuals in the third quartile of adherence to the Mediterranean diet (but not the highest) also showed better emotional wellbeing (OR = 1.32, 95% CI—1.06–1.65). However, these associations were more significant in women than in men. The Mediterranean diet demonstrated fewer statistically significant associations compared to other diet quality measures, possibly due to difficulties in adapting the Mediterranean diet framework to non-Mediterranean populations [53]. Another study conducted on 207 perimenopausal or menopausal Australian women, aged 40–60 years (mean age: 50.7 ± 4.3 years), aimed to explore the associations between adherence to the Mediterranean diet (assessed via the 14-item MEDAS questionnaire) and quality of life related to severity of menopausal symptoms. To do this, they used an 86-item self-administered online questionnaire, which was divided into four separate sections and included the 14-item MEDAS questionnaire and the SF-36. Participants reported low-to-moderate adherence to the Mediterranean diet (mean MEDAS score—5.2 ± 1.8, range 1–11). Multivariate regression analysis showed that adherence to the Mediterranean diet was not associated with severity of menopausal symptoms, but was positively associated with the physical function subscale of HRQoL (β = 0.173, 95% CI—0.001–0.029, *p* = 0.031). Furthermore, greater legume consumption (≥3 servings/week) was positively associated with physical function (β = 0.164, 95% CI—0.001–0.004, *p* = 0.04), and a lower intake of red and processed meat (≤1 serving/day) was linked to improved general health (β = 0.296, 95% CI—0.005–0.014, *p* < 0.001) [54].

However, some studies reported only marginal associations between adherence to the Mediterranean diet and HRQoL. A prospective study, conducted in Spain, investigated the association between the Mediterranean diet and HRQoL, involving two separate cohorts—the UAM-cohort, which included 2376 participants with a 3-year follow-up, and the Seniors-ENRICA cohort, which included 1911 participants, also male and female, with a 4 years of follow up. In order to obtain the dietary assessment, for the UAM-cohort they used a 14-item FFQ, which was used to develop an 8-item index of Mediterranean diet (UAM-MDP). In the Seniors-Enrica cohort, the dietary assessment was performed with computerized validated diet history, while the adherence to the Mediterranean diet was measured with the MEDAS score and the Trichopoulou’s MDS. HRQoL was measured with the SF-36 questionnaire (in the UAM cohort) and with the SF-12 questionnaire (in the Seniors-ENRICA cohort). In the UAM cohort, no significant associations were found between the UAM-MDP index and HRQoL; meanwhile, in the Seniors-ENRICA cohort, a higher MEDAS score was linked to a modest improvement in the physical component of the SF-36 (β = 1.34, 95% CI—0.21–2.47). However, neither the MEDAS nor the MDS scores were associated with either physical or mental component scores [55]. Also a cross-sectional study, conducted in Turkey, assessed the relationship between adherence to the Mediterranean diet and its effects on body composition, blood parameters and HRQoL in 142 adult volunteers (aged 18–65). To evaluate dietary habits, they used a seven-topic FFQ, while adherence to the Mediterranean diet was estimated using a 14-item Mediterranean diet adherence screener and the MDS. HRQoL was assessed using the SF-36 questionnaire. The mean Mediterranean diet adherence score was 6.89 out of 14, indicating low-to-moderate adherence. Participants with higher adherence scores exhibited better health outcomes than those with lower scores; furthermore, participants with higher adherence to the Mediterranean diet also showed lower fasting blood glucose (*p* = 0.041), lower triglycerides (*p* = 0.012) and lower insulin levels (*p* = 0.019). No significant correlations were found between Mediterranean diet adherence and most of the quality of life domains. An exception was the pain domain, wherein lower Mediterranean diet adherence corresponded to higher pain scores (*p* = 0.041) [56].

### 3.4. Details of the Main Results of the Studies Included: Patient Population

The main characteristics of the studies included in the systematic review reporting on the association between adherence to the Mediterranean diet and quality of life among the patient population are reported in Table 2. Most studies conducted on patients were cross-sectional analyses of patients with moderate-to-high cardiovascular risk. Among the largest studies, we can cite the analyses conducted on the baseline data of the PREDIMED-Plus, a Spanish multi-center randomized trial testing an energy-restricted Mediterranean diet combined with the promotion of physical activity and behavioral therapy for primary cardiovascular prevention compared to a Mediterranean diet alone. Researchers analyzed 6430 participants (age 55–70) with overweight/obesity and metabolic syndrome. Adherence to the Mediterranean diet was assessed using a 17-item questionnaire and the MDS. The association between Mediterranean diet adherence and HRQoL was evaluated using the SF-36 questionnaire. Higher adherence to the Mediterranean diet was significantly associated with better scores in all eight dimensions investigated in the SF-36. The largest differences (≥3 points) were observed for vitality (65.26 vs. 61.59), emotional role (87.92 vs. 84.36), and mental health (75.67 vs. 72.69) between the highest and lowest adherence quartiles. Improvements of at least 2 points were noted for physical functioning (77.53 vs. 75.16) and bodily pain (63.53 vs. 61.15). Adjusted multivariable regression models confirmed a positive linear association between Mediterranean diet adherence and HRQoL scores [57].

Among other studies involving patients with cardiometabolic risk factors, we can cite a cross-sectional study including 294 Spanish patients with T2DM (146 with diabetic retinopathy and 148 without retinopathy) in which dietary habits were evaluated using a semi-quantitative 101-item FFQ, while the adherence to the Mediterranean diet was estimated using the 18-point rMED; HRQoL was evaluated using the ADDQoL-19 questionnaire. Adherence to the Mediterranean diet (rMED ≥8) was positively associated with several HRQoL dimensions, such as self-confidence (SE = 0.428; *p* = 0.015), freedom to eat (SE = 0.839; *p* = 0.037), and freedom to drink (SE = 1.150; *p* = 0.015). However, a negative association was observed between intermediate rMED levels and ease of travel (SE = −0.450; *p* = 0.020). The analysis revealed no significant association between rMED and overall HRQoL scores [58]. Another study conducted in patients with T2DM involved 200 participants (mean age 55 years) recruited from the Internal Medicine Clinic of Famagusta State Hospital in the Turkish Republic of Northern Cyprus. Moderate adherence to the Mediterranean diet (assessed through the MEDAS score) was observed in 65.6% of male and 49.1% of female participants. Patients with moderate adherence demonstrated significantly higher physical functioning scores compared to those with low adherence. Using the SF-36 scale, HRQoL scores were moderate overall, with males reporting higher scores than females (*p* < 0.001), potentially linked to differences in diet adherence and lifestyle factors. Physical and mental health indicators were positively associated with adherence to the Mediterranean diet, highlighting the benefits of a Mediterranean dietary pattern [59]. A similar study conducted in Australia explored the association between adherence to a Mediterranean diet and subscales of HRQoL in 152 middle-aged and older adults with and without type-2 diabetes. All participants were community-dwelling, overweight or obese (BMI ≥ 25 kg/m^2^), and aged ≥50 years (T2DM cohort) or ≥60 years (non-T2DM cohort). Adherence to the Mediterranean diet was evaluated using the MEDAS, while HRQoL was measured using the SF-36 survey. The entire cohort’s mean MEDAS score was 5.3 ± 2.2, reflecting low-to-moderate adherence. Regression analyses revealed a significant positive association between Mediterranean diet adherence and the general health subscale for the entire cohort (β = 0.223, 95% CI—0.006, 0.044; *p* = 0.001) and the diabetic subgroup specifically (β = 0.280, 95% CI: 0.007, 0.054; *p* = 0.001). Meanwhile, no additional significant associations between HRQoL subscale and adherence to a Mediterranean diet were found [60]. In long-term at-risk patients, a cross-sectional study was conducted on 258 Spanish patients with type-1 diabetes (median age, 43 years). Dietary patterns were evaluated using the aMED, while HRQoL was assessed using the ADDQoL-19. Moderate or high adherence to the Mediterranean diet (aMED > 2) was significantly associated with greater diabetes-specific quality of life (β = 0.32, 95% CI = 0.03, 0.61, *p* = 0.029) [61]. A similar study has been conducted in another sample of 362 adults (ages 18–29) with T1DM in Andalusia, Spain, compiling an adaptation of the MEDAS and the Vida con Diabetes Tipo 1 (ViDa1) HRQoL questionnaire. In this study, higher adherence to the Mediterranean diet correlated with improved wellbeing and self-care. Multivariable regression analysis showed that insomnia had a greater impact on HRQoL than glycemic control or dietary habits, underscoring the importance of sleep assessment in diabetes management [62]. Another study from Spain on moderate CVD risk patients was conducted in the context of the MARK study, an investigation aiming to test the relation between diet quality and HRQoL in 314 individuals by administering a nine-item short questionnaire to assess the level of adherence to the Mediterranean diet and the SF-12 to evaluate HRQoL. The study showed that mental (r = 0.164, *p* < 0.01) and social functioning (r = 0.172, *p* < 0.01) and vitality (r = 0.122, *p* < 0.05) components of the HRQoL tool were related to the Mediterranean diet score, although in the multiple linear regression analysis, only the mental component score reached a significant association, with 1-point increase in the Mediterranean diet adherence score associated with a 1.2-point increase in the mental component score (*p* < 0.01) [63]. Concerning patients with already diagnosed CVD, a cross-sectional study investigated the association between adherence to the Mediterranean diet and HRQoL in 139 coronary artery disease (CAD) patients (mean age 63). Adherence to the Mediterranean diet was assessed using a 14-item questionnaire, and HRQoL was evaluated with the SF-36. A significant positive correlation was found between Mediterranean diet adherence and the physical component summary (PCS) and its subscales in the SF-36, as well as most mental component summary (MCS) subscales (excluding emotional role and social functioning) (*p* < 0.05). BMI, waist circumference, waist/height ratio, and body fat percentage were inversely correlated with PCS and MCS scores (*p* < 0.05) [64].

Some studies also investigated the potential role of the Mediterranean diet in the context of conditions different from cardiometabolic, yet still related to diseases with an inflammatory basis. A cross-sectional observational study investigated the association between adherence to the Mediterranean diet and HRQoL in 69 Greek patients with mild-to-severe psoriasis (35 men and 34 women) and 69 healthy controls matched for age, gender, BMI, and enrollment date. Adherence to the Mediterranean diet was assessed using the 12-item MDS, while HRQoL was evaluated using the DLQI. Psoriasis patients showed significantly lower adherence to the Mediterranean diet compared to controls (28.7 ± 4.4 vs. 33.9 ± 5.6, *p* < 0.001). Higher adherence to the Mediterranean diet was inversely associated with psoriasis risk (OR: 0.34, 95% CI: 0.13, 0.92, *p* = 0.03) [65]. Another cross-sectional study conducted in Greece aimed to explore the adherence to the Mediterranean diet and its association with asthma severity and HRQoL in 85 asthma patients (mean age 57 years). Dietary habits were evaluated with the 11-item MMDS, while HRQoL was calculated using the SF-12 survey and AQLQ. Most participants reported moderate adherence to the Mediterranean diet. Adherence to recommended levels of alcohol consumption within the Mediterranean diet was positively correlated with better PCS-12 scores (r = 0.437, *p* < 0.05). Multiple regression analysis revealed that asthma-specific quality of life (AQLQ score; β = 0.256, *p* = 0.008), adherence to Mediterranean alcohol consumption patterns (β = 0.220, *p* = 0.027), and lower BMI (β = −0.415, *p* < 0.001) significantly predicted better physical HRQoL. No significant differences were found in Mediterranean Diet adherence, AQLQ scores, or HRQoL scores between patients with mild and severe asthma, though the median PCS-12 score was lower in the severe asthma group (43.66 vs. 45.75; *p* = 0.076) [66]. Lately, a cross-sectional analysis conducted on 4470 participants (United States, both male and female, mean age—61.3 y) from the Osteoarthritis Initiative (OAI) evaluated the association between adherence to the Mediterranean diet and quality of life, pain, stiffness, disability, and depressive symptoms. Dietary pattern was analyzed with the use of a 70-item validated FFQ. The 11-item Mediterranean diet score was slightly modified to better capture the dietary habits of North Americans. HRQoL was measured using the SF-12, while physical and mental conditions were investigated via other specific tools. Participants with high adherence to the Mediterranean diet (highest quintile) showed significantly higher SF-12 scores (β = 0.10, 95% CI—0.05, 0.15; *p* < 0.0001) and better physical health. Furthermore, higher adherence to the Mediterranean diet was associated with a lower BMI and a lower prevalence of diabetes, as well as fewer depressive symptoms [67].

A group of studies investigated the role of adherence to the Mediterranean diet and HRQoL in patients suffering from inflammatory bowel diseases (IBDs). A cross-sectional study conducted in Turkey aimed to evaluate adherence to the Mediterranean diet in patients with IBD, including Crohn’s disease (CD) and ulcerative colitis (UC), and to examine its impact on disease activity and HRQoL. The study included 83 participants (age ≥ 18 years), who were diagnosed with IBD by the gastroenterologist and who were attending routine check-ups. The adherence to the Mediterranean diet of patients was assessed by face-to-face interviews conducted by a dietician and by the MEDAS, while HRQoL was measured using the SF-36. Higher Mediterranean diet adherence was associated with improved HRQoL in UC patients, particularly in the domains of emotional role functioning, mental health, and overall health perception (*p* < 0.05), while no significant associations were observed between Mediterranean diet adherence and disease activity or HRQoL in CD patients [68]. Another cross-sectional observational study examined the relationship between adherence to the Mediterranean diet and quality of life in 60 Greek patients with Crohn’s disease (22 men and 38 women). Adherence to the Mediterranean diet was assessed using the MDS, while HRQoL was evaluated using the CUCQ-8. Adherence to the Mediterranean diet was inversely correlated with disease activity (*p* = 0.039) and positively correlated with QoL (*p* = 0.046). The higher consumption of fruits, vegetables, and dairy products was notably associated with remission (*p* = 0.046, *p* = 0.001, *p* = 0.041, respectively) [69]. A similar study involving 86 patients with Crohn’s disease from Greece using the IBDQ to assess HRQoL reported a significant association between scores (β = 1.299, 95% CI—0.714–1.884, *p* = 0.030) [70]. Finally, another study conducted on 50 Greek patients with IBD compared to healthy controls using the EQ-5D-5L to assess HRQoL showed that IBD patients exhibited significantly lower HRQoL scores than healthy individuals (0.75 vs. 0.85, *p* < 0.05). While the Mediterranean diet showed a moderate correlation with HRQoL in healthy individuals (r = 0.131, *p* < 0.05), no significant association was observed among IBD patients [71]. In the context of bowel disorders, two surveys from the National Health and Nutrition Examination Survey (NHANES) (2009–2014), a representative sample of the USA, investigated the association between adherence to the Mediterranean diet (assessed via the alternate Mediterranean diet score) and HRQoL in both adults and teens with celiac disease compared to the whole samples (50 cases vs. 15,777 and 30 cases vs. 2296, respectively). Quality of life was assessed through age-specific tools (the CDQOL and CDPQOL), resulting in higher scores with increasing adherence to the Mediterranean diet only in teens but not in adults [72].

Some studies have been conducted to test the relation between adherence to the Mediterranean diet and HRQoL in cancer patients. A study conducted on the baseline data of 309 women within 12 months of diagnosis of breast cancer participating in the DediCa multicenter trial in Italy assessed HRQoL through three validated instruments measuring physical, mental and emotional and social factors (the European Organization for Research and Treatment of Cancer Quality of Life Questionnaire 3L, C30 and BR23). Higher adherence to the Mediterranean diet (PREDIMED score > 7) was observed in 55.7% of participants, and was associated with significantly higher physical functioning scores (*p* = 0.02) and lower pain scores (*p* = 0.04). Participants with higher adherence also reported better general wellbeing on the EQ-5D-3L questionnaire (*p* = 0.05). Multivariate regression analysis revealed significant positive associations between high Mediterranean diet adherence and physical function (β = 0.199; *p* = 0.001), while negative associations were identified with pain (β = −0.175; *p* = 0.002), insomnia (β = −0.114; *p* = 0.048), and dyspnea (β = −0.115; *p* = 0.045). Additionally, adherence was positively associated with the EQ-5D-3L score (β = 0.167; *p* = 0.004) [73]. Another study conducted on 68 Italian stage I-III breast cancer survivors assessed for HRQoL with the EORTC QLQ-C30 reported that patients with higher adherence to the Mediterranean diet showed a correlation between global health status and specifically emotional, cognitive, and social functioning, although the differences from lower-adherence groups were not significant [74].

Among studies conducted on end-stage organ diseases, one cross-sectional analysis conducted on 105 Greek patients (mean age 63.4 ± 13.1 years) with end-stage chronic kidney disease (CKD) assessed for adherence to the Mediterranean diet with the nine-item MDS and for HRQoL with the KDQOL-SF questionnaire reported no correlation of the diet with the overall HRQoL score or most subscales of the KDQOL-SF, except for the “work status” scale (r = 0.212, *p* < 0.05). Older patients and females experienced significantly lower HRQoL, with age inversely associated with the total HRQoL score (r = −0.394, *p* < 0.01). Educational and financial status were strong predictors of HRQoL, representing 28.1% of its variance (*p* < 0.001). Overweight participants scored higher on HRQoL compared to obese or normal-weight individuals [75]. Two multicenter studies investigated the HRQoL (assessed using the SF-12) in 511 liver transplant recipients (mean age—63.1 ± 10.8 years). Participants who were clinically stable and at least 12 months post-transplant were recruited from seven Italian Hepatology Units. Adherence to the Mediterranean diet was evaluated using the nine-item Medi-Lite score. Multivariate analysis revealed that higher adherence to the Mediterranean diet reduced the likelihood of impaired HRQoL (OR—0.84 for PCS-12, *p* = 0.001; OR—0.88 for MCS-12, *p* = 0.005). Occupation status and physical activity levels were also strong predictors of better physical health, with employed or moderately/highly active patients showing significantly higher PCS-12 scores (OR = 1.32, 95% CI—1.08–1.60) [76,77].

Three studies [78,79,80] have been conducted to evaluate the relation between diet and HRQoL in individuals with multiple sclerosis. A study enrolled 558 adults with multiple sclerosis (mean age—38.6 ± 12.2 years; 75.3% female) from diverse regions in Greece. Mediterranean diet compliance was assessed using the 11-item MDS and HRQoL was assessed through the MSQOL-54. Patients with higher Mediterranean diet adherence had higher HRQoL (RR = 1.95, 95% CI—1.71, 2.19), as well as a healthier BMI (RR = 2.13, *p* = 0.0018) and higher physical activity levels (RR = 1.76, *p* = 0.0037), and were less likely to have depression (RR = 2.08, *p* = 0.0107) [78]. Using the same sample (with an extended recruitment period), a subanalysis was conducted in 227 older adults with multiple sclerosis aged over 65 years. Also in this sub-group, higher Mediterranean diet adherence was significantly related with better cardiometabolic health parameters and biomarkers. Patients with greater adherence showed a 58% higher probability of achieving adequate physical activity and a two-fold greater probability of reporting better HRQoL (*p* = 0.0008). Multivariate logistic regression confirmed that higher Mediterranean diet adherence independently predicted better HRQoL (OR = 2.05, 95% CI—1.81, 2.28) as well as lower disease progression (OR = 2.21, *p* = 0.0002), healthier BMI (OR = 1.88, *p* = 0.0021), and greater muscle mass (mid-arm circumference ≥ 22 cm; OR = 1.28, *p* = 0.0198) [79]. Another study investigated the effect of the relationship between adherence to a Mediterranean diet and consumption levels of several food groups on HRQoL in 95 patients with multiple sclerosis aged 18–65 years. Dietary habits were estimated using an FFQ, while adherence to the Mediterranean diet was assessed using the MEDAS questionnaire. HRQoL was measured using the MSQOL-54. Adherence to the Mediterranean diet shows significant associations with both physical and mental health parameters of HRQoL. Specific food groups exhibited correlations with specific components of the MSQOL-54; daily fruit consumption was positively associated with mental health components, while vegetable consumption correlated with both physical and mental health scores, indicating broader benefits for HRQoL [80].

Only a few studies evaluated the HRQoL of patients over a follow-up period. A longitudinal study examined the relationship between adherence to the Mediterranean diet (assessed using the nine-point scale MDS) and the SF-36 in 50 patients with diabetes and chronic kidney disease (CKD) from Canada followed over a 5-year period. The average MDS was moderate (4.1 ± 1.6) and remained stable over time. Patients with CKD stages 3–5 had lower MDS scores compared to those in stages 1–2 (3.8 ± 1.5 vs. 4.6 ± 1.5; *p* < 0.001). Concerning HRQoL, higher MDS scores were associated with better mental health (84.4 ± 14.3 vs. 80.3 ± 17.1; *p* < 0.05) and general health scores (62.6 ± 21.0 vs. 56.3 ± 19.8; *p* < 0.001); higher MDS scores correlated with fewer depressive symptoms (9.1 ± 7.4 vs. 11.7 ± 10.6; *p* = 0.01). Lower MDS scores were linked to reduced kidney function. However, there was no significant relationship between MDS scores and HbA1c or lipid levels. Greater adherence to the Mediterranean diet was associated with higher dietary potassium intake (4.3 ± 1.6 vs. 3.6 ± 1.5 mg; *p* < 0.01) [81]. Another longitudinal examination of patients involved 78 Spanish morbidly obese participants undergoing bariatric surgery (Roux-en-Y gastric bypass or laparoscopic sleeve gastrectomy) over 12 months of follow-up. Adherence to the Mediterranean diet was measured using the 14-point MEDAS questionnaire, while HRQoL was assessed using the Moorehead–Ardelt Quality of Life Questionnaire II. The results indicate that participants who showed increased adherence to the Mediterranean diet achieved significantly higher total weight loss (mean %TWL—37.6%, 95% CI—35.5–39.8) compared to those who showed decreased or maintained adherence (mean %TWL—34.1%, 95% CI—31.8–36.5; *p* = 0.036). Similarly, excess weight loss was greater in the Mediterranean diet adherence group (71.2%, 95% CI—66.8–75.6) compared to the non-adherence group (63.1%, 95% CI—58.3–67.9; *p* = 0.018). HRQoL improved significantly across all domains compared to baseline, but there were no significant differences between groups with increased or decreased adherence to the Mediterranean diet or physical activity levels [82]. However, another study conducted on 56 obese patients from Spain assessed for HRQoL with the SF-36 and the MEDAS questionnaire to evaluate the adherence to the Mediterranean diet reported significant improvements in HRQoL, particularly in the physical component, and increased adherence to the Mediterranean diet (6.32 ± 1.9 to 7.62 ± 1.8, *p* < 0.001) at 18 months post-surgery. Patients who lost more than 25% of their total body weight showed greater improvements in both physical and mental HRQoL. Multivariable regression analysis identified total body weight loss as a significant predictor of improved physical HRQoL (*p* = 0.015), while improvements in Mediterranean diet adherence did not affect both physical and mental components of HRQoL [83].

**Table 2:** The main characteristics of the studies included in the systematic review reporting on the association between adherence to the Mediterranean diet and quality of life among the patient population.

Author, Year	Study Design	Study Name, Country (Follow-up)	Population, Number and Sex (Age)	Dietary Assessment	Mediterranean Diet Assessment	QoL Assessment	Main Findings
Alcubierre, 2016 [58]	Cross-sectional	Spain	294 MF with T2DM	Semi-quantitative 101-item FFQ	18-point rMED	ADDQoL-19	Higher adherence to the Mediterranean diet was positively associated with several HRQoL dimensions, such as self-confidence, freedom to eat, and freedom to drink, but no overall HRQoL scores.
Sanchez-Aguadero, 2016 [63]	Cross-sectional	MARK study, Spain	314 MF, intermediate CVD risk (35–74 y)	Not assessed	9-item short questionnaire	SF-12	Mental, social functioning, and vitality components were related to the Mediterranean diet total score. In multiple linear regression analysis for each 1-point increase in the Mediterranean diet adherence score, there was a 1.2-point increase in the mental component score.
Veronese, 2016 [67]	Cross-sectional	OAI, USA	4470 Knee osteoarthritis or high risk, MF	70-item FFQ	11-item aMED	SF-12	Higher SF-12 physical and mental health scores were shown by participants with higher adherence to the Mediterranean diet. Higher aMED was associated with better HRQoL and decreased pain, disability and depressive symptoms.
Galilea-Zabalza, 2018 [57]	Cross-sectional	PREDIMED-Plus study, Spain	6430 overweight or obese metabolic syndrome, M (55–75 y), F (60–75 y)	17-item questionnaire of adherence to a Mediterranean Diet	17-item Mediterranean diet screener	SF-36	Higher adherence to the Mediterranean diet was independently associated with significantly better scores in the eight dimensions of HRQoL. Adjusted differences of >=3 points were observed for vitality, emotional role and mental health between the grouops of highest and lowest adherence to the Mediterranean diet and of >=2 points for the other dimensions. Adherence to a Mediterranean diet and several dimensions of quality of life have shown a positive association.
Korovesi, 2019 [65]	Case-control	Greece	69 mild-to-severe psoriasis, 69 healthy controls, MF (47 y)	Not assessed	12-item MDS	DLQI	Adherence to the Mediterranean diet was independently and inversely associated with psoriasis occurrence, severity, and quality of life.
Özata Uyar, 2019 [64]	Cross-sectional	Turkey	139 coronary artery disease, MF	Not assessed	14-item MEDAS	SF-36	A significant positive correlation was reported between Mediterranean diet and all physical component summary (PCS, its subscales) and most mental component summary (MCS) (except emotional role and social health subscales) (*p* < 0.05). Inverse significant associations were observed between BMI, waist circumference, waist/height ratio, percent of body fat and both PCS and MCS (including most subscales).
Porciello, 2020 [73]	Cross-sectional	Italy	309 F with breast cancer (mean age 52.0 ± 9.2 y)	Not assessed	14-item PREDIMED	EQ-5D-3L, EORTC QLQ-C30, EORTC QLQ-BR-23	Higher adherence to the Mediterranean diet was positively associated with better physical functioning and wellbeing, and inversely associated with pain and insomnia.
Gils Contreras, 2020 [82]	Prospective	Spain (12 m)	78 people with obesity pending laparoscopic bariatric surgery, MF (18–66 y)	Not assessed	MEDAS	Moorehead–Ardelt Quality of Life questionnaire II.	Greater adherence to the Mediterranean diet following bariatric surgery was associated with greater weight loss in morbidly obese participants after bariatric surgery. However, neither the Mediterranean diet nor physical activity after surgery seems to have a direct influence on the HRQoL or food tolerance.
Granado-Casas, 2020 [61]	Cross-sectional	Spain	258 T1D, MF (>18 y)	101-item FFQ	10-item aMED	ADDQoL-19	Participants with T1D, and with a moderate and higher adherence to the Mediterranean diet, reported greater HRQoL.
Barchitta, 2020 [74]	Cross-sectional	Italy	68 breast cancer patients (36–68 y)	Not assessed	14-item MEDAS	EORTC QLQ-C30	Patients with higher adherence to the Mediterranean diet showed relations between global health status and specifically emotional, cognitive, and social functioning, although the differences from lower adherence groups were not significant.
Papada, 2020 [70]	Cross-sectional	Greece	86 patients with Crohn’s disease	Not assessed	11-item MDS	IBDQ	Higher adherence to the Mediterranean diet was correlated with IBDQ and disease activity.
Cordwell, 2021 [60]	Case–control	Australia	87 T2DM and 65 controls (>60 y)	Not assessed	14-item MEDAS	SF-36	Adherence to a Mediterranean diet was positively associated with the general health subscale of HRQoL, which was the most apparent in middle-aged to older adults with T2DM. Unable to rule out reverse causation. The T2DM cohort reported greater adherence to a Mediterranean diet compared with the non-T2DM cohort, although this was non-significant.
Picard, 2021 [81]	Prospective	Canada (5 y)	50 T1D/T2DM and stage 1–4 Chronic kidney disease, MF (18–80 y)	3-day food record	9-point MDS	SF-36, MDI	Adherence to the Mediterranean diet was overall moderate, though consistent over time, in patients living with diabetes and chronic kidney disease. No association between MDS and cardiometabolic risk factors; however, there was an association between lower MDS and lower kidney function. Higher MDS was associated with better mental and general health domains of HRQoL.
Floria, 2022 [75]	Cross-sectional	Greece	105 end-stage chronic kidney disease, MF (mean age 63.4 y)	Not assessed	9-item MDS	KD-QoL-SF	Adherence to the Mediterranean Diet was not correlated to the total QoL.
Gitto, 2022 [76]	Cross-sectional	Italy	511 liver transplant recipients, MF (≥18 y)	Not assessed	9-item MEDI-LITE	SF-12	The Mediterranean diet might help liver transplant recipients to improve their QoL.
Kontopoulou, 2023 [66]	Cross-sectional	Greece	85 asthma, MF, mean age 57 y	Not assessed	11-item MDS	AQLQ, SF-12	There was a significant correlation between moderate adherence to the Mediterranean diet and a relatively good level of HRQoL of the patients.
Çelik, 2023 [68]	Cross-sectional	Turkey	83 IBD, MF (≥18 y)	Not assessed	MEDAS	SF-36	Stronger adherence to the Mediterranean diet may help modulate disease activity and enhance HRQoL in ulcerative colitis patients, whereas its effects on Crohn’s disease remain inconclusive.
Kudret, 2023 [59]	Cross-sectional	Cyprus	200 T2DM, MF (30–65 y)	Not assessed	14-item MEDAS	SF-36	Participants with moderate adherence to the Mediterranean diet had higher physical function scores than those who had low adherence.
Cadenhead, 2023 [72]	Cross-sectional	NHANES (2009–2014), US	80 MF (adults and teens) with celiac disease and 15,777 adults and 2296 teens without celiac disease	Three 24 h recalls	9-item aMED	CDQOL, CDPQOL	Higher quality of life was found in teens with higher adherence to the Mediterranean diet, but not in adults.
Dakanalis, 2024 [78]	Cross-sectional	Greece	558 multiple sclerosis, MF (18–64 y)	Not assessed	11-item MDS	MSQOL-54	Higher Mediterranean diet adherence was related with a decreased disease disability symptoms’ intensity, as well as an improved HRQoL, a higher physical activity status, and fewer depressive symptoms.
Tryfonos, 2024 [79]	Cross-sectional	Greece	227 Multiple sclerosis, MF (>65 y)	Not assessed	11-item MDS	MSQOL-54	Higher Mediterranean diet compliance was independently associated with a lower prevalence of advanced disease progression, higher HRQoL, healthier BMI, and greater muscle mass.
Uygun Özel, 2024 [80]	Cross-sectional	Turkey	95 Multiple sclerosis, MF (18–65 y)	FFQ	14-item MEDAS	MSQOL-54	Adherence to the Mediterranean diet was associated with physical and mental quality of life parameters, independent of progression of disease. The HRQoL and disability level of multiple sclerosis patients was also associated with some food groups: daily fruit consumption was associated with mental health, and vegetable consumption was associated with both mental and physical components of the HRQoL score.
Migdanis, 2024 [69]	Cross-sectional	Greece	60 Crohn’s disease, MF (mean age 37.6 y)		11-item MDS	CUCQ-8	Patients with inactive Crohn’s disease demonstrated significantly greater adherence to the Mediterranean diet compared to those with active disease (*p* = 0.019). Moreover, adherence to this dietary pattern was inversely associated with disease activity (*p* = 0.039) and positively correlated with quality of life (*p* = 0.046). Notably, higher levels of consumption of fruits, vegetables, and dairy products was linked to disease remission (*p* = 0.046, *p* = 0.001, *p* = 0.041, respectively). The Mediterranean diet may contribute to modulating disease activity and enhancing the quality of life in patients with Crohn’s disease.
Christodoulou, 2024 [71]	Cross-sectional	Greece	338 of which 50 with IBD, MF		14-item MEDAS	EQ-5D-5L	The Mediterranean diet showed a moderate correlation with HRQoL in healthy individuals (r = 0.131, *p* < 0.05), while no significant association was observed among IBD patients.
Diaz-Gonzalez, 2024 [83]	Prospective	Spain	56 bariatric patients with obesity, MF (mean age 43.8 y)		14-item MEDAS	SF-36	Greater Mediterranean diet adherence did not affect both physical and mental components of HRQoL.
Nunez-Baila, 2024 [62]	Cross-sectional	Spain	362 T1DM, MF (18–29 y)		MEDAS	ViDa1	Higher adherence to the Mediterranean diet correlated with improved wellbeing and self-care. Multivariable regression analysis revealed that insomnia had a stronger influence on HRQoL compared to glycemic control or dietary habits.
Gitto 2024 [77]	Cross-sectional	Italy	511 liver transplant recipients, MF (≥18 y)	Not assessed	9-item MEDI-LITE	SF-12	Higher adherence to the Mediterranean diet was associated with a better physical component of quality of life.

Abbreviations: aMED, alternate Mediterranean Diet; ADDQoL-19, Audit of Diabetes-Dependent Quality of Life; AQLQ, Asthma Quality of Life Questionnaire; BMI, Body Mass Index; CDPQOL, Celiac Disease Pediatric-Specific Quality of Life; CDQOL, Celiac Disease-Specific Quality of Life; CVD, cardiovascular disease; DLQI, Dermatology Life Quality Index; FFQ, food frequency questionnaire; IBD, inflammatory bowel disease; IBDQ, inflammatory bowel disease questionnaire; MCS, Mental Component Summary; MDI, Major Depressive Inventory; MDS, Mediterranean Diet Score; MEDAS, Mediterranean Diet Adherence Screener; MDS, Mediterranean Diet Score; MSQOL-54, Multiple Sclerosis Quality of Life-54 Instrument; NHANES, National Health and Nutrition Examination Survey; OAI, osteoarthritis initiative; PCS, physical component summary; PREDIMED, Prevention with Mediterranean Diet; rMED, relative Mediterranean Score; SF-12, 12-Item Short-Form Health Outcome Survey; SF-36, Medical Outcome Study Short Form-36; T1DM/T2DM, Type 1/2 Diabetes Mellitus.

## 4. Discussion

In the present study, an overview of published reports exploring the association between adherence to the Mediterranean diet and various measures of HRQoL has been provided. Adherence to the Mediterranean diet was assessed through validated instruments in all studies. The measurement of HRQoL in adults and older individuals required the use of validated tools and scales that assess various dimensions of health. These included the physical domain (e.g., mobility, pain, fatigue), the psychological domain (e.g., mental health, anxiety, depression), and the social domain (e.g., relationships, social engagement, independence). Instruments, such as the SF-12 and SF36 were commonly employed to quantify HRQoL across different populations, although some specific tools were also implemented depending on the health status of the participants [84]. These measures allowed for the identification of specific areas wherein adherence to a healthy diet, such as the Mediterranean diet, could have had a stronger association. Most studies showed a significant association between adherence to the Mediterranean diet and HRQoL, with the most significant results retrieved for physical domains of quality of life. A few reports reported marginal findings, suggesting better physical health was associated with higher adherence to the Mediterranean diet, although not clearly statistically demonstrating this through specific HRQoL tools. Overall, diet seems to play a relevant role in both the general population and people affected by chronic conditions with an inflammatory basis.

The role of HRQoL in the adult population is a critical area of research, as improving health in older adults has profound implications not only for individual wellbeing, but also for reducing the economic and social costs associated with aging-related healthcare needs [85]. Physical resilience, the body’s ability to withstand, recover from, and adapt to physical stressors and health challenges, is a cornerstone of healthy aging and is crucial for individuals managing chronic diseases [86]. As people age, they naturally experience a decline in muscle mass, bone density, and metabolic efficiency, all of which contribute to frailty and functional decline [87]. However, maintaining or enhancing physical resilience through lifestyle interventions, particularly through diet and physical activity, can help mitigate these age-related declines and reduce the severity of chronic conditions [87]. Lifestyle choices, especially dietary patterns, are pivotal in strengthening the body’s resilience against the challenges of aging and chronic disease, and tailored nutritional strategies could be key in improving health outcomes, slowing disease progression, and enhancing the overall quality of life in older adults [88]. Ultimately, a lifestyle that includes both proper nutrition and regular physical activity is crucial for enhancing physical resilience in aging individuals [89]. By supporting muscle maintenance, bone health, metabolic function, and recovery, these lifestyle factors can reduce the risk of frailty and improve the prognosis for patients affected by chronic diseases [90]. Maintaining physical resilience through diet and activity allows older adults to remain active, independent, and better equipped to face the challenges that come with aging, ultimately improving their quality of life and reducing the burden of disease [87].

In light of such considerations and the results from this study, promoting healthy diets inspired by the Mediterranean diet principles could be a feasible strategy for primary and secondary prevention, and could reduce the burden of non-communicable diseases. Notably, its application in the clinical setting and the context of patients’ follow-up could also provide notable benefits. However, only a relatively low number of studies adopted the promotion of a healthy diet, such as the Mediterranean diet, in patients with non-communicable diseases. Some updated reports from the PREDIMED-plus elucidated the effects of a dietary intervention with a Mediterranean diet and its interaction with quality of life leading to improved cognitive outcomes (i.e., verbal memory and decision-making abilities) [91], as well as improved physical domain of HRQoL [92], potentially mediating the effects on weight loss. An earlier randomized controlled trial conducted in Spain examined the effects of a hypocaloric Mediterranean diet, with or without moderate-to-high-intensity exercise, on HRQoL, physical fitness, and metabolic syndrome risk factors in 40 sedentary adults (50–66 y) with metabolic syndrome. Quality of life assessments were measured by SF-36 and EuroQol EQ-5D questionnaires. The hypocaloric Mediterranean diet alone resulted in weight loss, reduced fat mass, decreased systolic and diastolic blood pressure, and improvements in fasting glucose, total cholesterol, and LDL-C levels (all *p* < 0.05). The combination of hypocaloric Mediterranean diet and periodized exercise sessions led to more substantial enhancements in these areas, along with improvements in additional HRQoL aspects, including physical role, pain perception, social functioning and overall health status. HRQoL domains such as vitality, physical function, and general health improved in both groups, but only the combined group showed significant benefits in bodily pain, social function and physical role limitations (all *p* < 0.05) [93]. A parallel-arm, open-label intervention trial aimed to compare the effects of long-term versus short-term Mediterranean diet adherence on skin microvascular function and quality of life. There were two groups of participants; the first was a control group of long-term Mediterranean diet adherers, recruited in Greece, while the second was an intervention group of non-adherers, from the UK, who followed a four-week Mediterranean diet intervention. HRQoL was estimated with the WHOQoL-BREF. At the end of the study, both groups demonstrated high Mediterranean diet adherence. Regarding HRQoL domains, the physical health domain improved significantly in the intervention group (13.7 ± 1.2 vs. 15.9 ± 1.2; *p* < 0.05), while social domain scores were higher in the control group. No significant differences were detected in lipid profiles between the groups or between baseline and post-intervention phases [94]. Finally, a randomized controlled trial conducted in Mexico, involving 130 patients with rheumatoid arthritis (female only), aimed to investigate the effects of a dynamic exercise program (DEP) combined with a Mediterranean diet on HRQoL. Participants were randomly assigned to one of four groups—Mediterranean diet + DEP (n = 36), DEP only (n = 37), Mediterranean diet only (n = 40) or a control group (n = 31). Dietary habits were assessed using 24 h food recall. HRQoL was evaluated with the SF-36. At the end of the intervention, the Mediterranean diet + DEP group demonstrated significant improvements in the global HRQoL score (+15.3 points), surpassing both the DEP-only and Mediterranean diet only (+3.5 points) groups, while the control group exhibited a decline (−4.6 points; *p* = 0.01). Regarding the physical component, the Mediterranean diet + DEP group showed the largest increase (+15.5 points), followed by the DEP-only group (+12.5 points) and the MD-only group (+5.1 points), whereas the control group decreased (−1.7 points; *p* = 0.03) [95].

There are several mechanisms providing a robust rationale for using the Mediterranean diet as a potential tool to improve HRQoL, especially in older individuals. The anti-inflammatory and antioxidant effects of the Mediterranean diet, particularly its rich content of (poly)phenols, omega-3 polyunsaturated fatty acids (PUFA), and antioxidant vitamins, play a key role in reducing the chronic low-grade inflammation and oxidative stress that characterize many aging-related diseases [96,97]. Chronic inflammation has been linked to the pathogenesis of a wide range of pathological conditions, including autoimmune inflammatory disorders, cardiometabolic and neurodegenerative diseases [98]. The diet’s rich antioxidant profile, primarily derived from plant-based foods like fruits, vegetables, and olive oil, has also been implicated also in reducing oxidative stress, a key contributor to cellular damage and aging [99]. Oxidative stress leads to the accumulation of reactive oxygen species (ROS) that can damage cellular components, including lipids, proteins, and DNA, thereby accelerating aging and promoting the development of age-related diseases. Antioxidants, such as the (poly)phenols found in red wine, berries, and olive oil, scavenge these ROS, thereby protecting cells from oxidative damage and promoting healthier aging [100,101]. This effect is particularly important to preventing vascular abnormalities, where oxidative stress plays a critical role in disease progression [102,103,104].

The Mediterranean diet’s capacity to modulate inflammatory pathways offers significant potential in preventing non-communicable diseases and improving HRQoL [105]. Specifically referring to some compounds, omega-3 PUFA and certain (poly)phenols highly represented in fruits and vegetables (but also nuts, red wine, and olive oil) have been shown to lower levels of pro-inflammatory cytokines such as TNF-α and IL-6, and to inhibit the expression of inflammatory mediators involved in various non-communicable diseases [106]. The Mediterranean diet’s potential to enhance gut health also plays a crucial role in its therapeutic effects. The Mediterranean diet’s emphasis on high-fiber foods such as vegetables, whole grains, and legumes, combined with healthy fats from olive oil and nuts, helps regulate the gut microbiome, promoting the growth of beneficial bacteria and reducing harmful microbial populations [107]. This microbiota modulation has been deemed responsible for the local and systemic activation of the immune system, with resulting effects on gut and cardiovascular health [108,109,110,111]. Moreover, the reduction in systemic low-grade inflammation and positive microbiome regulation may also lead to improvements in insulin sensitivity and insulin resistance, both hallmarks of T2DM [112,113,114]. The beneficial effects of the Mediterranean diet on weight management, another key factor in diabetes control, are also noteworthy, as the diet encourages consumption of nutrient-dense, satiating foods that may help prevent obesity [115,116].

One of the most compelling areas of research is the impact of the Mediterranean diet on neurodegenerative diseases, particularly Alzheimer’s and Parkinson’s diseases. These disorders, which are characterized by progressive cognitive decline and motor dysfunction, respectively, are highly prevalent in aging populations and represent a significant burden on quality of life [117,118,119]. Evidence suggests that adherence to the Mediterranean diet may slow the onset and progression of cognitive decline in patients with mild cognitive impairment and Alzheimer’s disease [32]. Components of the Mediterranean diet, such as the consumption of (poly)phenol-rich foods (such as phenolic acids, resveratrol, and specific flavonoids) and omega-3 PUFA (predominantly found in fish but also oily seeds), may contribute to enhanced cognitive function by reducing neuroinflammation and oxidative stress [120,121]. Current evidence shows that higher intakes of (poly)phenols [122,123] and omega-3 PUFA [124,125] are associated with a lower risk of cognitive decline and dementia. Additionally, these compounds have been shown to improve brain plasticity, support mitochondrial function, and modulate the gut–brain axis, all of which are implicated in maintaining cognitive health [126,127,128,129].

Beyond its anti-inflammatory and antioxidant effects, the Mediterranean diet also influences other important biological mechanisms involved in aging and disease progression. Diet plays a central role in supporting physical health, and may be a contributing factor to quality of life in older individuals [130,131]. An optimal diet comprises all nutrients required for muscle maintenance, bone health, immune function, and energy metabolism to support an active lifestyle [132,133]. Such an adequate intake of macro- and micronutrients, as well as a healthy gut microbiome, are crucial for maintaining muscle mass and function, especially in older adults [134]. The scientific literature supports the hypothesis that higher adherence to the Mediterranean diet is associated with better physical fitness among younger individuals [135]. Concerning physical and muscular health, specific minerals and vitamins, such as vitamin D, calcium, and magnesium are important for maintaining bone density and preventing osteoporosis [136,137]. Moreover, diets rich in anti-inflammatory foods, such as those high in omega-3 PUFA, found in fatty fish and nuts, can help modulate the chronic low-grade inflammation that can impair the body’s ability to repair tissues, hinder muscle regeneration, and promote the development of frailty [138,139].

Concerning the role of the Mediterranean diet in cancer, the aforementioned antioxidant and anti-inflammatory mechanisms are central to its potential beneficial effects [140]. The rich array of bioactive compounds it contains, including (poly)phenols, flavonoids, carotenoids, and vitamin C, all of which possess potent antioxidant properties, may help mitigate oxidative stress, which is a fundamental contributor to DNA damage and the initiation of carcinogenesis [141]. By scavenging ROS and neutralizing free radicals, the Mediterranean diet may lower the risk of mutation accumulation in cells, a key step in cancer development [19,142]. Additionally, the anti-inflammatory properties of these bioactive compounds can modulate inflammatory pathways that are often dysregulated in cancerous tissues, reducing the chronic inflammation that fuels tumor progression and metastasis [97,143]. The Mediterranean diet also influences key molecular pathways involved in cellular growth and apoptosis, further contributing to its potential anticancer properties. Studies suggest that the diet’s high content of monounsaturated fats, especially oleic acid derived from olive oil, may suppress the activity of pro-inflammatory mediators such as NF-kB and COX-2, both of which are implicated in cancer initiation and progression [144]. Moreover, the polyphenols found in olive oil, such as oleuropein and hydroxytyrosol, have been shown to inhibit angiogenesis, the process by which tumors form new blood vessels to sustain their growth, thereby limiting tumor expansion [145]. Beyond its direct effects on cancer cells, the Mediterranean diet also promotes the modulation of the immune system; by enhancing gut microbiota diversity, which is vital for immune homeostasis, the diet may strengthen the body’s natural defense mechanisms against cancer [146]. In the context of cancer patients, adherence to the Mediterranean diet has been shown to yield significant improvements in both treatment efficacy and side-effect management [146]. Cancer therapies, particularly chemotherapy and radiation, often induce severe side effects, including fatigue, nausea, loss of appetite, and weight loss, which can drastically reduce a patient’s quality of life [147]. The anti-inflammatory and antioxidant effects of the Mediterranean diet may help mitigate these adverse reactions, promoting better treatment tolerance and enhancing recovery [148]. Moreover, the nutrient-dense nature of the Mediterranean diet, which supports the optimal intake of vitamins, minerals, and essential fatty acids, may help preserve muscle mass and prevent cachexia, a condition commonly associated with advanced cancer [149].

The Mediterranean diet has been shown to exert positive effects on mood regulation and mental health [150]. The aforementioned mechanisms concerning neurodegenerative disorders involving antioxidants, (poly)phenols, and omega-3 fatty acids can also modulate the brain’s neurochemistry by reducing oxidative stress and inflammation, factors known to contribute to mood and affective disorders [151]. Moreover, the Mediterranean diet’s role in promoting a healthy, balanced lifestyle extends beyond its individual nutrients to its broader impact on overall lifestyle factors. The traditional Mediterranean lifestyle encourages mindful eating, portion control, and social eating practices, which can improve both physical and mental aspects related to quality of life [152]. The regular consumption of healthy meals, combined with social engagement, conviviality, and daily physical activity, contributes to a holistic approach to aging that fosters longevity and better HRQoL [153]. The Mediterranean diet’s emphasis on social eating practices, particularly in Mediterranean cultures, is associated with stronger social interactions and a greater sense of community [154]. Regular communal meals, often centered around the preparation and sharing of healthy, fresh foods, foster social bonds and improve social support networks, which in turn positively influence mental health and wellbeing [155,156]. The social dimension of dietary patterns and lifestyle habits may play a pivotal role in enhancing psychological health, which is a key component of overall quality of life [157]. The Mediterranean diet encourages mindful eating, which involves greater attention to food quality, preparation, and the eating experience [158,159]. This approach to eating fosters healthier relationships with food, reduces instances of overeating, and encourages greater satisfaction with meals, all of which can positively impact HRQoL by enhancing individuals’ physical and emotional satisfaction [160]. These factors contribute to better HRQoL by fostering physical and emotional wellbeing. The synergistic relationship between diet, physical activity, and social interaction in the Mediterranean lifestyle supports a holistic approach to wellbeing, promoting a sense of balance, contentment, and overall life satisfaction. Research has shown that Mediterranean dietary patterns, when coupled with regular physical activity, are associated with lower levels of depression, improved mood, and enhanced cognitive function, all of which contribute to better health outcomes in aging individuals [161]. However, factors such as eating schedules, eating locations, and the inclusion of culturally accepted foods have been only rarely investigated in the context of HRQoL, yet are considered relevant to the overall value of the Mediterranean diet intended as a lifestyle beyond its nutritional features [162].

The prevalence of non-communicable diseases is on the rise with the prevalence of suboptimal diets, characterized by a high consumption of energy-dense foods, rich in trans fats and free sugars, and at the same time low in fiber and essential nutrients [163]. While evidence from the observational studies indicates that higher adherence to healthy diets, such as the Mediterranean diet, contributes to the prevention of chronic diseases and relates to better quality of life, several other studies report potential detrimental effects of suboptimal diets, such as a Western diet. A study from the SUN cohort exploring the association between a posteriori derived dietary patterns and HRQoL showed that baseline adherence to a Western dietary pattern, rich in red meats, processed pastries and fast-food, was inversely associated with self-perceived quality of life after 4 years of follow-up [164]. Such a relation was corroborated in several other studies conducted among the general population reporting that adherence to a more Westernized diet related to worse HRQoL [165,166]. Similar results were observed among cancer patients. The results from the ColoCare Study showed that adhering to a Western dietary pattern after surgery was inversely associated with HRQoL among colorectal cancer patients [167], and subsequently the Western diet was concurrently associated with a component of HRQoL [168]. A study conducted among Korean breast cancer survivors reported that the Western dietary pattern was marginally significantly associated with the components of physical functioning [169]. Also, women with osteoporotic disorders adherent to a Western diet reported lower quality of life when compared to those adherent to a prudent diet [170]. These results further support the hypothesis that a balanced diet may play a role in promoting better quality of life in both healthy individuals and patients affected by diseases.

The application of a Mediterranean-type diet in non-Mediterranean countries holds significant potential for improving public health outcomes, yet it presents a range of implications, barriers, and possibilities that must be carefully considered. The positive health outcomes related to higher adherence to the Mediterranean diet could be particularly impactful in regions experiencing rising rates of obesity, metabolic syndrome, and other diet-related illnesses [171]. However, the cultural and culinary divergence between the traditional Mediterranean food pattern and the typical diets of non-Mediterranean populations may represent a major barrier for its application. Many countries outside the Mediterranean region have deeply ingrained food traditions, and dietary shifts may face resistance due to taste preferences, food availability, and local agricultural practices [172]. The specific ingredients on which the Mediterranean diet relies, such as olive oil, fresh seafood, and a variety of fruits and vegetables, may not always be accessible or affordable in these regions, making widespread adoption economically challenging [173]. Furthermore, the food industry in non-Mediterranean countries often promotes highly processed, convenience-oriented foods, which are not only nutritionally inferior but also more in line with prevailing consumer behavior, adding another layer of difficulty in promoting the Mediterranean diet [174]. Despite these barriers, there are significant possibilities for adapting and promoting the Mediterranean diet in non-Mediterranean settings. Public health initiatives could focus on local adaptations of the Mediterranean food pattern, incorporating region-specific ingredients while maintaining the core principles of the diet, such as plant-based foods, healthy fats, and moderate fish consumption [175]. Additionally, educational campaigns emphasizing the health benefits of the Mediterranean diet, along with cooking classes and community programs, could increase awareness and facilitate dietary shifts [176]. Governments could also play a key role by supporting agricultural policies that encourage the cultivation of Mediterranean diet-friendly crops, such as fruits, vegetables, and legumes, which may also benefit local economies [177]. Moreover, the growing interest in sustainable and environmentally friendly diets presents an opportunity to position the Mediterranean diet as a model for promoting both human and planetary health [178]. Ultimately, while the challenges are considerable, the potential to improve global health outcomes through the promotion of a Mediterranean-type diet is substantial, and with thoughtful, culturally sensitive adaptations, it could become a viable dietary model in non-Mediterranean countries [179].

The results presented in this study should be considered while taking into account certain limitations. First, most studies had a cross-sectional design; hence, the retrieved associations between adherence to the Mediterranean diet and HRQoL cannot be attributed to causality. Moreover, although some studies provided analyses of potential confounding factors, such as physical activity level or socioeconomic and cultural status, one cannot rule out the possibility of residual confounding originating from such aforementioned variables, or unmeasured or unknown factors (i.e., genetics). Second, although the instruments used to assess the level of adherence to the Mediterranean diet were validated, in some cases, they have been modified to be better applied to the specific sample (i.e., non-Mediterranean population) or due to constraints of the FFQ (i.e., lacking key food items). Moreover, the tools used in the various studies reviewed to evaluate HRQoL were not identical, thus results cannot be directly compared. Finally, the majority of the included studies have been conducted in the Mediterranean region, thus limiting the generalizability of the retrieved results to other non-Mediterranean populations.

## 5. Conclusions

In conclusion, adherence to the Mediterranean diet offers significant benefits in the management of various chronic diseases commonly encountered in aging populations. Its multifaceted mechanisms, including anti-inflammatory, antioxidative, metabolic-regulating, and gut-modulating effects, underscore its potential use as a therapeutic tool for improving health in patients with neurodegenerative diseases, metabolic disorders, and musculoskeletal conditions. Furthermore, the diet’s ability to promote a healthier lifestyle and reduce the burden of chronic disease may offer a sustainable and effective strategy for enhancing aging individuals’ health and quality of life, ultimately contributing to more effective and less invasive treatments for chronic diseases. As research continues to elucidate the precise molecular and physiological pathways through which the Mediterranean diet exerts its beneficial effects, its role in clinical practice is poised to expand, offering patients a powerful dietary intervention for disease management and healthy aging.

## Figures and Tables

**Table 1:** The main characteristics of the studies included in the systematic review reporting on the association between adherence to the Mediterranean diet and quality of life among the general population.

Author, Year	Study Design	Study Name, Country (Follow-up)	Population, Number and Sex (Age)	Dietary Assessment	Mediterranean Diet Assessment	QoL Assessment	Main Findings
Munoz, 2009 [38]	Cross-sectional	Spain	3054 MF (25–74 y) and 6322 MF (35–88)	165-item FFQ	9-item MDS	SF-12	The age-adjusted linear regression analysis revealed a significant direct association of the MDS with self-reported mental and physical health in both sexes.
Henrìquez Sànchez, 2012 [39]	Prospective	SUN cohort, Spain (4 y)	11,015 MF (interquartile 27–45 y)	136-item FFQ	9-item MDS	SF-36	Adherence to the Mediterranean diet was directly associated with self-perceived mental and physical quality of life. This association was stronger for the physical domains.
Bonaccio, 2013 [46]	Cross-sectional	Moli-sani study, Italy	16,937 MF (>35 y)	188-item FFQ, PCA, FAC	9-item MDS, 11-item IMI	SF-36	Adherence to a Mediterranean diet pattern is associated with better HRQoL. Stronger association with mental health than with physical health. Neither the antioxidant content of diet nor the dietary fiber intake influenced the association between Mediterranean scores and physical health.
Milte, 2015 [53]	Prospective	WELL study, Australia (2 y)	2457 MF (55–65 y)	111-item FFQ	8-item MDS (no olive oil in FFQ)	RAND-36	Higher MDS was correlated with HRQoL after two years. In women, higher adherence to the Mediterranean diet was linked to enhanced general health and energy, while higher overall diet quality was connected to better emotional wellbeing and improved physical functioning.
Pèrez-Tasigchana, 2015 [55]	Prospective	UAM cohort (3 y) and Seniors-ENRICA (4 y), Spain	2376 MF and 1911 MF (>60 y)	Diet history	8-item UAM-MDP, 14-item PREDIMED score, 9-item MDS	SF-36, SF-12	After follow-up, no relevant association was found between higher adherence to the Mediterranean diet and better HRQoL.
Zaragoza-Martì, 2018 [40]	Cross-sectional	Spain	351 MF (>60 y)	MEDIS-FFQ	9-item MDS	SF-12	Mediterranean diet adherence was linked to HRQoL. Participants with greater adherence to the Mediterranean diet were more physically active, had better HRQoL and consumed less alcoholic beverages. The adherence to the Mediterranean diet was directly associated with self-perceived physical and mental function in both men and women, as well as with life satisfaction among women.
Godos, 2019 [47]	Cross-sectional	Italy	1937 MF (18–90 y)	110-item FFQ	Medi-Lite	MANSA	A significant linear trend of association was reported for the overall quality of life and adherence to the Mediterranean diet. All the domains of the MANSA, except for self-rated mental health, were individually associated with higher adherence to the Mediterranean diet.
Pano, 2020 [45]	Prospective	SUN cohort, Spain	15,674 MF (mean age 38.5 y)	136-item FFQ	9-item MDS	SF-36	Higher adherence to the Mediterranean diet was associated with all components of HRQoL (global, physical, mental, and transition).
Apostolaki 2021 [49]	Cross-sectional	MINOA, Greece	436 MF (>65 y)	Not assessed	11-item MDS	SF-36	Higher Mediterranean diet adherence were associated with higher ratings in the Feeling of Safety/Trust and Value of Life and Social Agency indicators, but not the rest of the Social Capital indicators; instead, for Mediterranean diet adherence and health, we observed the contrary, as improved scoring in seven out of eight parameters of the HRQoL were associated with higher Mediterranean diet adherence (except for the role of emotional health parameter). Stronger positive relationship between physical health and Mediterranean diet adherence than for mental health. Total social capital, physical component summary and value of life and social agency were confirmed by multivariate linear regression as independent predictors of Mediterranean diet adherence.
Kalkuz, 2021 [56]	Cross-sectional	Turkey	142 MF (18–65 y)	229-item FFQ	14-item MEDAS	SF-36	Participants with greater adherence to the Mediterranean diet and better serum markers than those with low adherence. No significant correlation found between adherence to the Mediterranean diet and HRQoL.
Ribot-Rodriguez, 2022 [44]	Cross-sectional	Spain	17,333 MF (>18 y)	Not assessed	14-item PREDIMED	SF-12	A higher Mediterranean Diet adherence and better physical component of the SF-12 were observed in the participants with healthy cardiometabolic status, unlike in the diseased ones. Additionally, greater Mediterranean diet adherence was observed in participants with very good health compared to those with poor/fair HRQoL. Europeans/Caucasians showed a greater Mediterranean diet adherence score and better PCS12 score than participants with other ethnicities.
Lòpez-Olivares, 2023 [41]	Cross-sectional	Spain	127 MF (29–67 y)	Not assessed	14-item MEDAS	SF-36	An improved HRQoL was associated with Mediterranean diet adherence. Vitality, physical functioning, physical role, bodily pain, the mental component summary, and the Mediterranean diet adherence score increased when WHR and BMI decreased. Higher adherence to the Mediterranean diet was also associated with a better cardiometabolic profile and improved emotional states.
Lòpez-Olivares, 2023 [42]	Cross-sectional	Spain	399 MF	Not assessed	14-item MEDAS	SF-36	The physical health component of HRQoL was significantly associated with higher Mediterranean diet adherence scores.
Mantzorou, 2023 [50]	Cross-sectional	Greece	3254 MF (>65 y)	Not assessed	11-item MDS	SF-36	High adherence to the Mediterranean diet was independently associated with better HRQoL.
Godos, 2023 [48]	Cross-sectional	Italy	883 MF (>50 y)	110-item FFQ	Medi-Lite	MANSA	Higher adherence to the Mediterranean diet was associated with better HRQoL alongside lower odds of cognitive impairment.
Pavlidou, 2023 [51]	Cross-sectional	Greece	3721 MF (18–65 y)	Not assessed	11-item MDS	WHO-QoL-Bref	Positive association between Mediterranean diet adherence and improved lifestyle factors, better anthropometric profiles and enhanced mental health outcomes during COVID-19 pandemic.
Flor-Alemany, 2024 [52]	Prospective	GESTAFIT, Spain, HealthyMoms, Sweden	138 F and 302 F (32.9 y and 31.3 y)	78-item FFQ; 3-day 24-h recall	8-item Mediterranean diet adherence score	SF-36	A greater Mediterranean diet adherence during pregnancy was associated with lower bodily pain in both Mediterranean and Non-Mediterranean populations especially in the third trimester of pregnancy, within which women generally experience a deterioration in HRQoL.
Conde-Pipo, 2024 [43]	Cross-sectional	Spain	520 MF (41–80 y)	Not assessed	14-item MEDAS	SF-36	In individuals aged 71–80, a lower adherence to the Mediterranean diet correlated with a deterioration in self-perceived physical health. This decline was primarily attributed to the insufficient intake of key dietary components such as fresh vegetables, legumes, fish, and fruit.
Byrne-Kirk, 2024 [54]	Cross-sectional	Australia	207 perimenopausal and menopausal F (40–60 y)	Not assessed	14-item MEDAS	SF-36	No significant association between adherence to a Mediterranean-lifestyle diet and the severity of menopausal symptoms but was positively associated with the physical function subscale of HRQoL.

Abbreviations: BMI, Body Mass Index; FAC, food antioxidant content; FFQ, food frequency questionnaire; GESTAFIT, Gestation and Fitness Project; IMI, Italian Mediterranean diet Index; MANSA, Manchester Short Assessment of Quality of Life; MEDAS, Mediterranean Diet Adherence Screener; MEDIS-FFQ, Mediterranean Diet Score Short Food Frequency Questionnaire; MDS, Mediterranean Diet Score; MINOA, Mediterranean Diet in Older Adults; PCA, principal component analysis; PREDIMED, Prevention with Mediterranean Diet; SF-12, 12-Item Short-Form Health Outcome Survey; SF-36, Medical Outcome Study Short Form-36; SUN, Seguimento Universidad de Navarra; T1DM/T2DM, Type 1/2 Diabetes Mellitus; UAM-MDP, Mediterranean Dietary Pattern Index; WELL, Wellbeing, Eating, and Exercise for a Long Life; WHOQOL-BREF, World Health Organization’s Quality of Life Questionnaire brief version; WHR, waist/hip ratio.

## Data Availability

Data sharing is not applicable to this article as no data were created or analyzed in this study.

## Supplementary Materials

The following supporting information can be downloaded at: https://www.mdpi.com/article/10.3390/nu17030577/s1, Table S1: The Meta-analysis of Observational Studies in Epidemiology (MOOSE) guidelines. Table S2: Search strategy. Table S3: PICOS criteria. Table S4: Assessment of study quality according to the Newcastle–Ottawa Quality Assessment Scale for cross-sectional studies. Table S5: Assessment of study quality according to the Newcastle–Ottawa Quality Assessment Scale for case–control studies. Table S6: Assessment of study quality according to the Newcastle–Ottawa Quality Assessment Scale for prospective studies.

## Abbreviations

The following abbreviations are used in this manuscript:

ACT	Asthma Control Test
ADDQoL-19	Audit of Diabetes-Dependent Quality of Life
AHT	Anti-Hypertension Medication
AQLQ	Asthma Quality of Life Questionnaire
BMI	Body Mass Index
CAD	Coronary artery disease
CDQOL	Celiac Disease-Specific Quality of Life
CDPQOL	Celiac Disease Pediatric-Specific Quality of Life
CKD	Chronic kidney disease
CUCQ-8	Crohn’s and Ulcerative Colitis Questionnaire
CVD	Cardiovascular disease
DEP	Dynamic exercise program
DLQI	Dermatology Life Quality Index
DTSQ-s	Diabetes Treatment Satisfaction Questionnaire-status version
EDSS	Expanded Disability Status Scale
EQ-5D-3L	European Quality of Life 5 Dimensions 3 Level
FFQ	Food Frequency Questionnaire
GESTAFIT	Gestation and Fitness Project
HRQoL	Health-related quality of life
IBD	Inflammatory bowel disease
IBDQ	Inflammatory Bowel Disease Questionnaire
IMI	Italian Mediterranean Diet Index
IPAQ	International Physical Activity Questionnaire
LDL-c	LDL-cholesterol
MANSA	Manchester Short Assessment of Quality of Life
MCS	Mental Component Summary
MEDAS	Mediterranean Diet Adherence Screener
MEDIS-FFQ	Mediterranean Diet Score Short Food Frequency Questionnaire
MetS	Metabolic Syndrome
MDS	Mediterranean Diet Score
MINOA	Mediterranean Diet in Older Adults
MOOSE	Meta-Analyses of Observational Studies in Epidemiology
MSQOL-54	Multiple Sclerosis Quality of Life-54 Instrument
OAI	Osteoarthritis initiative
PCA	Principal component analysis
PCS	Physical component summary
PREDIMED	Prevention with Mediterranean Diet
PUFA	Polyunsaturated fatty acids
rMED	relative Mediterranean Score
RAND-36	RAND 36-Item General Health Survey
RFS	Recommended Food Score
ROS	Reactive oxygen species
SF-12	12-Item Short-Form Health Outcome Survey
SF-36	36-item Short Form Health Outcome Study
SUN	Seguimento Universidad de Navarra
SWLS	Satisfaction with Life Scale
T1D/T2DM	Type 1/2 Diabetes Mellitus
UAM-MDP	Mediterranean Dietary Pattern Index
WELL	Wellbeing, Eating and Exercise for a Long Life
WHR	Waist/hip ratio
WHOQoL-BREF	World Health Organization Quality of Life Brief Version
